# Increased gene sampling strengthens support for higher-level groups within leaf-mining moths and relatives (Lepidoptera: Gracillariidae)

**DOI:** 10.1186/1471-2148-11-182

**Published:** 2011-06-24

**Authors:** Akito Y Kawahara, Issei Ohshima, Atsushi Kawakita, Jerome C Regier, Charles Mitter, Michael P Cummings, Donald R Davis, David L Wagner, Jurate De Prins, Carlos Lopez-Vaamonde

**Affiliations:** 1 Department of Entomology, University of Maryland, College Park, MD, USA; 2 Division of Evolutionary Biology, National Institute for Basic Biology, Okazaki, Japan; 3 Center for Ecological Research, Kyoto University, Kyoto, Japan; 4 Institute for Bioscience and Biotechnology Research, University of Maryland, College Park, MD, USA; 5 Laboratory of Molecular Evolution, Center for Bioinformatics and Computational Biology, University of Maryland, College Park, MD, USA; 6 Department of Entomology, Smithsonian Institution, Washington, D.C., USA; 7 Department of Ecology & Evolutionary Biology, University of Connecticut, Storrs, CT, USA; 8 Royal Museum for Central Africa, Tervuren, Belgium; 9 INRA, UR0633 Zoologie Forestière, F-45000, Orléans, France

## Abstract

**Background:**

Researchers conducting molecular phylogenetic studies are frequently faced with the decision of what to do when weak branch support is obtained for key nodes of importance. As one solution, the researcher may choose to sequence additional orthologous genes of appropriate evolutionary rate for the taxa in the study. However, generating large, complete data matrices can become increasingly difficult as the number of characters increases. A few empirical studies have shown that augmenting genes even for a subset of taxa can improve branch support. However, because each study differs in the number of characters and taxa, there is still a need for additional studies that examine whether incomplete sampling designs are likely to aid at increasing deep node resolution. We target Gracillariidae, a Cretaceous-age (~100 Ma) group of leaf-mining moths to test whether the strategy of adding genes for a subset of taxa can improve branch support for deep nodes. We initially sequenced ten genes (8,418 bp) for 57 taxa that represent the major lineages of Gracillariidae plus outgroups. After finding that many deep divergences remained weakly supported, we sequenced eleven additional genes (6,375 bp) for a 27-taxon subset. We then compared results from different data sets to assess whether one sampling design can be favored over another. The concatenated data set comprising all genes and all taxa and three other data sets of different taxon and gene sub-sampling design were analyzed with maximum likelihood. Each data set was subject to five different models and partitioning schemes of non-synonymous and synonymous changes. Statistical significance of non-monophyly was examined with the Approximately Unbiased (AU) test.

**Results:**

Partial augmentation of genes led to high support for deep divergences, especially when non-synonymous changes were analyzed alone. Increasing the number of taxa without an increase in number of characters led to lower bootstrap support; increasing the number of characters without increasing the number of taxa generally increased bootstrap support. More than three-quarters of nodes were supported with bootstrap values greater than 80% when all taxa and genes were combined. Gracillariidae, Lithocolletinae + *Leucanthiza*, and *Acrocercops *and *Parectopa *groups were strongly supported in nearly every analysis. *Gracillaria *group was well supported in some analyses, but less so in others. We find strong evidence for the exclusion of Douglasiidae from Gracillarioidea sensu Davis and Robinson (1998). Our results strongly support the monophyly of a G.B.R.Y. clade, a group comprised of Gracillariidae + Bucculatricidae + Roeslerstammiidae + Yponomeutidae, when analyzed with non-synonymous changes only, but this group was frequently split when synonymous and non-synonymous substitutions were analyzed together.

**Conclusions:**

1) Partially or fully augmenting a data set with more characters increased bootstrap support for particular deep nodes, and this increase was dramatic when non-synonymous changes were analyzed alone. Thus, the addition of sites that have low levels of saturation and compositional heterogeneity can greatly improve results. 2) Gracillarioidea, as defined by Davis and Robinson (1998), clearly do not include Douglasiidae, and changes to current classification will be required. 3) Gracillariidae were monophyletic in all analyses conducted, and nearly all species can be placed into one of six strongly supported clades though relationships among these remain unclear. 4) The difficulty in determining the phylogenetic placement of Bucculatricidae is probably attributable to compositional heterogeneity at the third codon position. From our tests for compositional heterogeneity and strong bootstrap values obtained when synonymous changes are excluded, we tentatively conclude that Bucculatricidae is closely related to Gracillariidae + Roeslerstammiidae + Yponomeutidae.

## Background

Researchers conducting molecular phylogenetic studies are frequently faced with the decision of what to do when weak branch support is obtained for key nodes of importance. As one solution, the researcher may choose to sequence additional orthologous genes of appropriate evolutionary rate. Indeed, it is well known that increasing the number of characters can improve branch support (e.g. [1-5]). However, generating large, complete data matrices can become increasingly difficult as the number of characters increases. Two empirical studies [6,7] have concluded that augmenting genes even for a subset of taxa can improve branch support. However, because each study differs in the number of characters and taxa, there is still a need for additional studies that examine whether incomplete sampling designs are likely to aid at increasing deep node resolution.

In this paper, we target Gracillariidae, a Cretaceous-age (~100 Ma) group of leaf-mining moths [8] to test whether the strategy of adding genes for a subset of taxa can improve branch support for deep nodes. Gracillariidae, with 1,855 species [9,10], is one of the largest groups of leaf-mining Lepidoptera with numerous economically important species that cause agricultural damage [9,11-16]. Gracillariids show a diversity of life-history strategies, such as fruit mining, stem mining, leaf rolling, boring, and galling [11,17], and some species change strategies during development [17-20]. Despite the agricultural importance and diversity of life-history strategies, the systematics of Gracillariidae is poorly understood. Monophyly of the superfamily Gracillarioidea as currently defined by Davis and Robinson [11] remains uncertain. The phylogenetic position of Gracillarioidea in Lepidoptera is also relatively unclear, though recent molecular studies strongly support a close relationship to Yponomeutoidea [7,21,22].

Davis and Robinson's classification includes four families in Gracillarioidea, Bucculatricidae, Douglasiidae, Gracillariidae, and Roeslerstammiidae. Bucculatricidae and Douglasiidae were included in Gracillarioidea based on nine morphological features that they share with Gracillariidae and Roeslerstammiidae, including two from the larva, two from the pupa, and five from the adult [11]. Others have included Bucculatricidae, Gracillariidae, and Phyllocnistidae (the latter now in Gracillariidae [9,10,23,24]), Bucculatricidae, Gracillariidae, and Lyonetiidae [25], or Bucculatricidae, Gracillariidae and Roeslerstammiidae [26]. While some putative relationships have been postulated for higher-level relationships within Gracillarioidea based on morphology (e.g. [24,27], the trees presented in these studies were based on phenetic similarity rather than discrete character analysis. The only phylogenetic study that examined higher-level gracillariid relationships was a recent study aimed at resolving broader relationships of Lepidoptera that included 14 gracillarioid species [22]. The authors suggested that Gracillarioidea might not include Bucculatricidae or Douglasiidae. Most phylogenetic studies within Gracillarioidea have focused mainly at the genus level or below (e.g. host races of *Acrocercops transecta *[28,29], *Epicephala *and relatives [30-32], and *Phyllonorycter *[33,34]).

**Figure 1:**
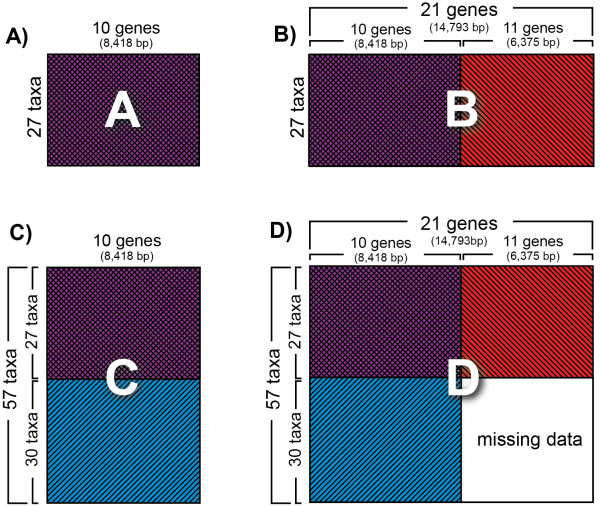
**Four data sets representing different taxon/gene sampling strategies**. A) 27 taxa × 10 genes, B) 27 taxa × 21 genes, C) 57 taxa × 10 genes, D) combination of B and C into a single data set with a block of missing data accounting for approximately a quarter of total data.

This study utilizes 21 nuclear protein-coding genes to evaluate the effect of augmenting sequence data for a subsample of taxa and to tackle the problem of the phylogeny of Gracillariidae and their relatives. Fifty-seven taxa, including exemplars representing the major lineages of Gracillarioidea plus outgroups, were initially sequenced for ten genes (8,418 bp). After discovering that many deep divergences within the superfamily could not be recovered with strong branch support, we sequenced 11 additional genes (6,375 bp) for 27 taxa representing the major lineages of Gracillarioidea (21 genes total, 14,793 bp). We compared results from four data sets differing in gene and taxon sampling design (Figure 1), to assess whether one design can be favored over another. We also examined the effect of excluding synonymous changes, which at deeper levels in our taxon sample are subject both to saturation and to divergence in base composition, possibly obscuring phylogenetic signal.

## Methods

### Taxon sampling

The present study included 45 species of Gracillarioidea, of which 39 were Gracillariidae (Additional file 1). Taxa were chosen to represent the major lineages as defined by the classification of Davis and Robinson [11]. Whenever possible, we included the type species or genus. Twelve outgroups were chosen based on the availability of sequence data and their phylogenetic proximity to Gracillarioidea in two recent molecular phylogenetic studies of ditrysian Lepidoptera [7,21]. While we mention Oecophyllembiinae in our discussion, we follow Davis and Robinson [11] and Vári et al. [35] and do not formally recognize this subfamily.

### Gene sampling

Ten nuclear protein-coding genes, totalling an alignment length of 8,418 bp, were initially chosen for this study (Table 1). These genes were included because they had some of the highest amplification success rates and had proven useful for estimating a "backbone" phylogeny of Lepidoptera (see http://www.leptree.net/) and are among the 68 gene regions originally developed for deep-level phylogenetics of Arthropoda [36]. We created two data sets for ten genes, one with 27 taxa (data set A, Figure 1A) and another with 57 taxa (data set C, Figure 1C). After discovering that ten genes did not adequately resolve phylogenetic relationships among subfamilies, we chose a resource-efficient approach to tackle the problem of weak branch support for deep nodes in the tree. We additionally sequenced eleven genes for 27 taxa and combined this additional sequence data with the original ten (data set B, 14,793 bp; Figure 1B). These additional eleven genes were chosen based on amplification success and examining the average rate of non-synonymous change from a previous study [36]. Throughout the paper, we refer to the "ten genes" and "eleven genes" as the original ten and additional eleven genes. Because our primary goal was to produce the best estimate for relationships of Gracillariidae and relatives, we combined data sets A through C to create data set D (57 taxa × 21 genes, 14,793 bp; Figure 1D). We assessed the differences in tree topology and branch support among these data sets and tested the effect of synonymous changes on each.

**Table 1:** Representation of genes and their amplicon names in each of the four data sets.

			Data set			
			
			A	B	C	D
**Gene**	**Amplicon name and reference**	**Length** **(bp)**	**10 g × 27 t**	**21 g × 27 t**	**10 g × 57 t**	**21 g × 27 t**

40fin2_3	Phosphogluconate dehydrogenase [36]	750		X		X
42fin1_2	Putative GTP-binding protein [36]	840		X		X
109fin1_2	Gelsolin [36]	552	X	X	X	X
192fin1_2	Glutamyl- & prolyl-tRNA sybphetase [36]	402		X		X
197fin1_2	Triosephosphate isomerase [36]	444		X		X
262fin1_2	Proteasome subunit [36]	501		X		X
265fin2_3	Histidyl-tRNA sybphetase [36]	447	X	X	X	X
268fin1_2	AMP deaminase [36]	768	X	X	X	X
3007fin1_2	Glucose phosphosphate dehydrogenase [36]	621	X	X	X	X
3017fin1_2	Tetrahydrofolate sybphase [36]	594		X		X
3070fin4_5	Alanyl-tRNA sybphetase [36]	705		X		X
8028fin1_2	Nucleolar cysteine-rich protein [36]	324		X		X
8091fin1_2	Glucose phosphate isomerase [36]	666		X		X
acc2_4	Acetyl-coA carboxylase [36]	501	X	X	X	X
CAD	Pyrimidine biosynthesis [85]	2913	X	X	X	X
DDC	Dopa-decarboxylase [86]	708	X	X	X	X
EF-1alpha	Elongation factor-1 alpha [37]	519	X	X	X	X
enolase	Enolase [87]	1134	X	X	X	X
histone 3	Histone 3 [38]	273	X	X	X	X
period	Period [88]	747		X		X
wingless	Wingless [89]	402		X		X

A box with an "X" indicates a gene that was included in that particular data set.

Specifically, data set A (27 taxa × 10 genes) was constructed to examine whether modest taxa and gene sampling can strongly resolve relationships of Gracillariidae and relatives. Data set B (27 taxa × 21 genes) was constructed to examine whether nearly doubling the number of characters would boost branch support for deep splits that were not well supported with ten genes. Data set C (57 taxa × 10 genes) was built to assess the effects of adding more taxa to data set A. Finally, data set D (57 taxa × 21 genes) was constructed to examine how trees generated from a data set that included all taxa and genes (but also approximately a quarter of the characters without data) compared to those from the other three data sets. The amount of missing data for each of the four data sets (A through D) was 13.6%, 14.3%, 31.2%, and 43.6% respectively.

For all genes except elongation factor-1 alpha (EF-1α [37]) and histone 3 (H3 [38]), nucleic acid sequences were generated from mRNAs amplified with RT-PCR following laboratory protocols, primer sequences, and amplification strategies of Regier et al. [39]. For EF-1α and H3, we followed methods outlined in Kawakita et al. [30], Kawakita and Kato [40], Ogden and Whiting [38], and amplified directly from genomic DNA. Each single-gene data set was individually translated and aligned with the "Translation Align" option in Geneious 5.1 [41] after making sure the data set began with the first codon position (nt1). The alignment was visually inspected, and checked twice for frame-shifts and the presence of termination codons. Difficult to align regions were assessed in GBlocks 0.91b [42] and removed as they can cause problems in phylogeny estimation [43,44]. Sequences were also assessed for contamination and sample-switching error, by generating pairwise distance tables for nt12, nt3, and nt123 in PAUP* 4b10 [45] and ML bootstrap trees in GARLI 1.0 [46] for each gene before all genes were concatenated. The 21 data matrices were concatenated with Geneious [41] and the entire edited sequence data set visually checked. GenBank accession numbers are listed in Additional file 1.

### Phylogenetic analysis

Phylogenetic analyses were conducted with maximum likelihood (ML) as implemented in GARLI 1.0 [46] and GARLI-PART 0.97 [47]. All settings were kept as default except where indicated below. We used jModelTest [48] to determine the best substitution model for each data set, which in nearly all cases was the General-Time-Reversible (GTR) model [49,50], with among-site rate heterogeneity modeled according to a gamma (Γ) distribution [51] while allowing for a proportion of invariable sites (I) [52]. Two thousand ML and bootstrap tree searches were conducted for analyses that applied a nucleotide substitution model. We also ran codon model analyses [53] as implemented in GARLI. Due to computational limitations at the time of this study, each codon analysis was conducted with 100 ML tree searches and 100 bootstrap replicates. To expedite tree searches, we used Grid computing [54] through The Lattice Project [55]. For consistency in the characterization of results, we will refer to bootstrap support (BP) of 70-79% as "moderate," support ≥ 80% (but < 90%) as "strong," and ≥ 90% as "very strong." We use the arbitrary cutoff of 80% BP as a measure to compare the number of nodes with strong support across individual genes.

### Base compositional heterogeneity

Base compositional bias can cause unrelated lineages to incorrectly group together (e.g. [56-60]). While models for phylogenetic analysis assume compositional homogeneity, strong compositional bias is common at sites capable of undergoing synonymous substitution [21,36,61]. In order to examine the effect of compositional heterogeneity, we examined five different character partitions, with and without synonymous change: (a) "nt123": all nucleotides and all changes; (b) "codon": all nucleotides and changes, but implementing a codon model, which "down-weights" synonymous changes because of their relatively rapid evolution; (c) "degen1" [62,63]: all sequence sites with the potential to undergo synonymous changes fully degenerated, an extension of the RY coding scheme of Phillips et al. [64]; (d) "partitioned": all nucleotides partitioned into mostly synonymously evolving and mostly non-synonymously evolving sites, specifically, the partition, "noLRall1 + nt2" versus "LRall1 + nt3" of Regier et al. [62]; and (e) amino acids. As an alternative means to filter synonymous substitutions, in some cases we also analyzed the noLRall1 + nt2 data set alone (see Discussion).

To further investigate the potential influence of compositional heterogeneity, we conducted chi-square tests of among-taxon heterogeneity on data set B (27 taxa × 21 genes). We chose data set B because it includes the largest number of characters (14,793 bp) with a relatively low percentage of missing data (14.3%) out of the four data sets. Chi-square tests were conducted on a character set undergoing mostly synonymous change, nt3, and one undergoing mostly non-synonymous change, noLRall1 + nt2. We conducted the test for various groups in Gracillariidae and outgroups on both the entire character set, and after eliminating invariable sites in the degen1 data set. To gauge the possible effect of compositional heterogeneity on phylogeny inference, we compared neighbor-joining trees using two different distances: ML distances based on the GTR model, which can be influenced by compositional heterogeneity; and Euclidean distances calculated on the proportions of the four nucleotide states treated as independent characters, which will reflect only compositional heterogeneity. Compositional distances were generated using a Perl script that was written with modification of the MBE Toolbox [65] and calculated with PAUP* [45].

### Testing alternative hypotheses

Morphology and larval mining patterns predict the monophyly of Gracillariidae + Bucculatricidae + Roeslerstammiidae [26], Gracillariinae + Lithocolletinae [24], and Oecophyllembiinae + Phyllocnistinae [11,35,66], but some of these proposed higher-level groups were not recovered. To test whether these differences between morphological (and behavioral) versus molecular inferences were "real," i.e. not attributable to sampling error in the molecular data, we used the Approximately Unbiased (AU) test of Shimodaira [67]. The AU test ranks trees and determines if trees under a topological constraint describe the data significantly worse than the best tree.

To compare the confidence between our results and prior morphology-based hypotheses, we conducted separate analyses in which groups believed to be monophyletic were constrained. ML trees were calculated with constraints enforced, and the ML tree from the constrained and the unconstrained analyses compared with the AU test. Each analysis applied the same number of ML runs determined to be appropriate for that character partition as described above. Site likelihoods were estimated with PAUP* [45]. For each data set, we combined the site likelihoods generated from all ML constraint analyses together into a single file with the unconstrained site likelihoods. In CONSEL 0.1j [68], the AU test statistic of Shimodaira [67] was used to determine the difference in fit to data of the constrained and unconstrained trees.

## Results

### Gene versus taxon sampling

Sampling design had the greatest influence on the recovery and bootstrap support of deep nodes in Gracillariidae and relatives, which was especially pronounced for the G.B.R.Y. clade (Gracillariidae + Bucculatricidae + Roeslerstammiidae + Yponomeutidae). Bootstrap support for this clade rose for all five analytical methods when the number of sampled characters was nearly doubled (data set A [27 taxa × 10 genes] versus B [27 taxa × 21 genes]; Table 2). Degen1 provided the strongest support for the G.B.R.Y. clade, rising from 74% (data set A) to 90% (data set B). An increase was also seen when we analyzed the complementary 11 gene, 27 taxa data set (data set B minus A), which had 84% BP for the G.B.R.Y. clade (data not shown). Conversely, doubling the number of taxa in data set A, yielding data set C, lowered support from 74% to < 50% BP for degen1 (data set A versus C; Table 2). Augmenting data set C with sequence data for 11 genes for just over half the number of taxa greatly improved branch support (all five analyses resulted in > 50% BP; data set C versus D, Table 2). There was little difference in bootstrap support for the G.B.R.Y. clade between the two data sets with the greatest amount of gene sampling (data set B versus D). Bootstrap support for other deep, non-G.B.R.Y. clades changed very little.

**Table 2:** Bootstrap support values across data sets for selected clades.

Data set	Analysis	'G.B.R.Y.' clade	Gracillariidae + Bucculatricidae + Yponomeutidae ('G.B.Y.' clade)	Gracillariidae + Roeslerstammiidae + Yponomeutidae ('G.R.Y.' clade)	Gracillariidae + Yponomeutidae ('G.Y.' clade)	Gracillariidae	Lithocolletinae + *Leucanthiza*	*Acrocercops *group	*Gracillaria *group	*Parectopa *group	Phyllocnistinae + Oecophyllembiinae + *Dendrorycter *+ *Marmara*
A	nt123	[< 50]	[< 50]	< 50	< 50	99	N/A	87	N/A	100	N/A
	codon	[< 50]	[< 50]	[< 50]	[< 50]	99	N/A	86	N/A	100	N/A
	degen	74	55	[< 50]	[< 50]	100	N/A	99	N/A	100	N/A
	partitioned	[< 50]	[< 50]	[< 50]	[< 50]	99	N/A	89	N/A	100	N/A
	aa	[< 50]	[< 50]	[< 50]	[< 50]	97	N/A	90	N/A	100	N/A

B	nt123	[53]	[< 50]	< 50	[< 50]	100	N/A	98	N/A	100	N/A
	codon	[54]	[< 50]	< 50	[< 50]	100	N/A	93	N/A	100	N/A
	degen	90	[< 50]	< 50	[< 50]	100	N/A	92	N/A	100	N/A
	partitioned	[62]	[< 50]	[< 50]	[< 50]	100	N/A	94	N/A	100	N/A
	aa	66	[< 50]	[< 50]	< 50	98	N/A	97	N/A	100	N/A

C	nt123	[< 50]	[< 50]	[< 50]	[< 50]	99	100	98	71	100	[< 50]
	codon	[< 50]	[< 50]	[< 50]	[< 50]	99	100	98	58	100	[< 50]
	degen	[< 50]	[< 50]	[< 50]	[< 50]	100	100	100	89	100	[< 50]
	partitioned	[< 50]	[< 50]	[< 50]	[< 50]	99	100	97	77	100	[< 50]
	aa	[< 50]	< 50	[< 50]	[< 50]	90	100	93	< 50	100	[< 50]

D	nt123	[55]	[< 50]	< 50	[< 50]	99	100	100	67	100	51
	codon	[61]	[< 50]	< 50	< 50	97	100	100	100	100	< 50
	degen	83	[< 50]	< 50	< 50	100	100	100	93	100	[< 50]
	partitioned	[59]	[< 50]	< 50	[< 50]	99	100	97	67	100	51
	aa	75	[< 50]	[< 50]	< 50	89	100	94	< 50	100	< 50

Square brackets indicate support values for clades that were not present in the ML tree.

Gracillariinae was polyphyletic in all analyses conducted (data sets A through D), and the position of Phyllocnistinae remains unclear, as it was ancestral to the Lithocolletinae + *Leucanthiza *in data set D, but was sister to the *Parectopa *group in data sets A through C for degen1 and amino acids. For nt123 and codon analyses, the Phyllocnistinae was sister to the *Parectopa *group (nt123, data sets A, C), sister to *Dendrorcyter *+ *Marmara *(nt123, data set D; codon, data sets C, D), or *Eumetriochroa *(nt123, data set C, codon, data sets A, B). Partitioned analyses ("noLRall1 + nt2" versus "LRall1 + nt3") gave the same placement for the Phyllocnistinae as did nt123 in all four data sets.

### Agreement and conflict among individual genes

There were no strongly supported groups that conflicted with each other across genes, and few nodes above the subfamily level were moderately or strongly supported by any one gene alone. Instances of strong bootstrap support by only one gene were: 83% for Gracillariidae (CAD), 96% for the *Acrocercops *group (CAD), and 82% for *Eumetriochroa *+ Phyllocnistinae (*Period*; Additional file 2).

### Base compositional heterogeneity

Results of the chi-square tests for compositional heterogeneity are shown in Table 3. Homogeneity was not rejected for any group in the noLRall1 + nt2 character set. In contrast, nt3 showed highly significant heterogeneity across all taxa and the five taxon subsets. As a gauge of the possible misleading signal produced by compositional heterogeneity, we calculated neighbor-joining trees on distances reflecting only composition for nt123 and nt3. In these trees, Bucculatricidae clustered with five other gracillarioid and non-gracillarioid taxa that are together separated by long internal branches from the Tineidae and the remaining species in the tree (Additional file 3).

**Table 3:** Results of Chi-square tests on nucleotide compositional homogeneity.

	*P *value for character set	
	
Taxon (number of species)	noLRall1 + nt2	nt3
All (27)	> 0.999	< 0.001
Gracillariidae (11)	> 0.999	< 0.001
Oecophyllembiinae *sensu *Kumata + Phyllocnistinae (3)	0.969	< 0.001
Bucculatricidae + Tineidae (3)	0.953	< 0.001
Bucculatricidae + Outgroups + *Klimeschia *- Tineidae (10)	>0.999	< 0.001
Outgroups + *Klimeschia *- Tineidae (9)	> 0.999	< 0.001
		
Total number of characters	8701	4937

### Relationships of Gracillariidae and Gracillarioidea

**Figure 2:**
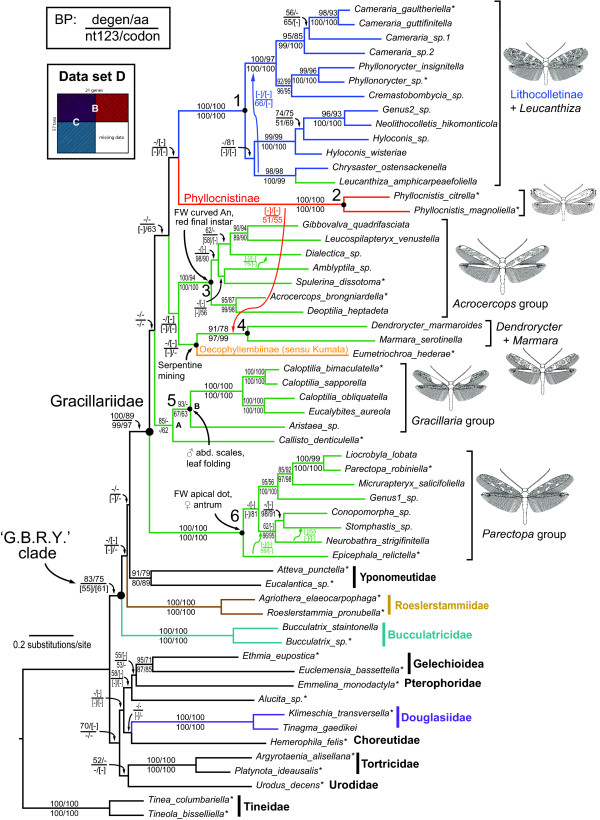
**Maximum likelihood degen1 tree for data set D**. Large numbers denote six major clades in Gracillariidae (see Results). Asterisks indicate taxa sequenced for 21 genes. Hyphens denote support values < 50%. Square brackets, shown only for nodes with support > 50% that conflict with the nt123 ML tree, denote groupings not present in the ML tree for that analysis. Green branches lead to taxa placed in Gracillariinae. Morphological and behavioral traits that are characteristic of each group are also noted.

All analyses resulted in a polyphyletic Gracillarioidea *sensu *Davis and Robinson [11]; specifically, Douglasiidae was consistently separated from Bucculatricidae, Gracillariidae, and Roeslerstammiidae by two or more nodes. Monophyly of the superfamily was significantly rejected at *P *≤ 0.015 in six of eight AU tests (Table 4). In all analyses, branch support for the monophyly of Gracillariidae was robust (≥ 97% BP, Table 2). In general, nt123, codon and nt123 partitioned results were similar in topology and branch support, while degen1 and amino acids were similar to each other, but differed from the other results in topology. For data set D (57 taxa × 21 genes), degen1 resulted in a monophyletic 'G.B.R.Y.' clade with strong bootstrap support (83%, Figure 2). The G.B.R.Y. clade was never recovered in nt123, codon and nt123 partitioned ML trees, but the bootstrap consensus from these analyses supported the G.B.R.Y. clade with weak, but evident signal (up to 62% BP, Table 2). The latter three methods resulted in Bucculatricidae diverging before all taxa except the designated outgroup, Tineidae (e.g. Additional files 4, 5, 6).

**Table 4:** Results of Approximately Unbiased (AU) significance tests [67] for non-monophyly of predicted clades for data sets C and D.

	*P *values: data sets C/D			
	
Predicted clade	nt123	codon	degen	AA
Gracillarioidea *sensu *Davis & Robinson	**0.015/0.002**	**0.007/0.006**	0.083/<**0.001**	0.081/**0.008**
Gracillariinae + Lithocolletinae	**0.011/0.002**	**0.013/0.011**	0.455/0.161	0.152/0.119
Gracillariinae	**< 0.001/< 0.001**	**< 0.001/< 0.001**	**< 0.001/< 0.001**	**< 0.001/< 0.001**
Gracillariinae minus *Leucanthiza*	**0.015/0.001**	**0.021/< 0.001**	0.107/0.084	0.104/0.202
Oecophyllembiinae *sensu *Kumata + Phyllocnistinae	0.467/0.165	0.385/0.739	0.339/0.352	0.472/0.073

nt123, all nucleotides; Codon, codon model; degen, degeneracy1; AA, amino acids. Groups that were significant at alpha = 0.05 are shown in bold.

While there was some support for the G.B.R.Y. clade, none of the analyses provided > 55% BP for any inter-family relationships within this clade. To assess the possible sister group of Gracillariidae, we examined whether the ML trees consistently recovered Gracillariidae plus any combination of Bucculatricidae, Roeslerstammiidae, or Yponomeutidae. ML analyses recovered a 'G.R.Y.' clade eight times, a 'G.Y.' clade five times, and a 'G.B.Y.' clade two times (Table 2). No 'G.B.', 'G.R.', or 'G.B.R.' clades were found in any of the best ML trees.

Within Gracillariidae, there was strong support for 28 of the 36 total nodes (78%) with data set D, and nearly every species was placed into one of six lineages that are either monotypic or strongly supported in our sampling. These lineages, labeled on Figure 2, include: 1) Lithocolletinae + *Leucanthiza*, 2) Phyllocnistinae (excluding *Eumetriochroa*), and four clades within Gracillariinae: 3) *Acrocercops *group, 4) *Dendrorycter *+ *Marmara*; 5) *Gracillaria *group, and 6) *Parectopa *group. Kumata compared the morphology of these groups and recognized that each has unique features absent in others (e.g. [69-72], some noted in Figure 2).

Branch support within these six gracillariid clades was strong. There was > 90% BP for eight of the eleven nodes within Lithocolletinae + *Leucanthiza*, three of five nodes within the *Acrocercops *group, five of six nodes in the *Parectopa *group, and all nodes in the *Gracillaria *group. Monophyly of the subfamily Gracillariinae Stainton 1854, as previously defined, was not recovered, and statistically rejected in all eight AU tests (*P *< 0.001, Table 4). Rejection of gracillariine monophyly was also evident even when *Leucanthiza *was excluded from Gracillariinae in the AU tests (see Gracillariinae minus *Leucanthiza*, Table 4). Monophyly of Gracillariinae + Lithocolletinae, as previously proposed by Kuznetzov and Stekol'nikov [24] was rejected by nt123 and codon model analyses (*P *< 0.05). Monophyly cannot be rejected for the sister-group relationship of the Oecophyllembiinae (*sensu *Kumata) + Phyllocnistinae, however, as *P *> 0.073 under the AU test in all cases (Table 4). While we do not formally designate any new taxonomic names in this study, we recognize these well-supported clades as the first step toward a phylogenetic reclassification of Gracillariidae.

## Discussion

### Augmentation of sequence data and its effect on branch support

Partial augmentation of gene sampling can improve estimates of deep relationships of Gracillariidae (comparison of data set C and D), as it increased support for the G.B.R.Y. clade for all five character treatments, most strikingly for degen1 (a BP increase from < 50% to 83%). While partial or full augmentation of gene sampling generally improved branch support for deep nodes, other nodes at the superfamily level are not robustly supported even with > 14 kb of sequence data (Table 2). Short internal branches for deep divergences and the difficulty of achieving strong support for some nodes even with 21 genes may reflect a rapid radiation, which likely characterizes many divergences among lepidopteran families [21,22,73] and other insect orders [74].

**Figure 3:**
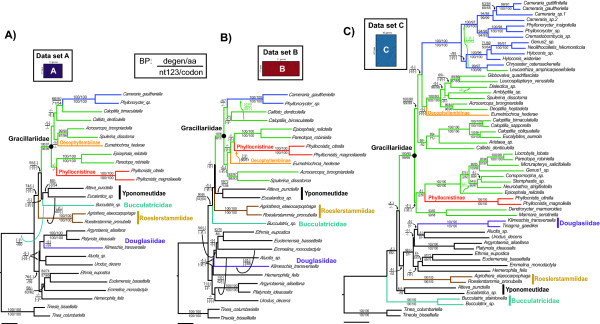
**ML trees based on non-synonymous differences only (degen1) of data sets A through C**. Bucculatricidae + Gracillariidae + Roeslerstammiidae + Yponomeutidae form a monophyletic group for data sets A and B. Scale bar = 0.02 substitutions/site.

The amount of missing data in data set D, accounting for roughly a quarter of the total matrix, does not apparently induce phylogenetic artifacts of the kind postulated by Lemmon et al. [75]. For degen1, the problematic Bucculatricidae is left out of the G.B.R.Y. clade in the ML tree from non-augmented data set C (Figure 3C), but partially augmented data set D pulls it into the G.B.R.Y. clade. We favor the topology from the partially augmented data set D over the non-augmented data set C, as the close relationship of Bucculatricidae to Gracillariidae corroborates morphological [24,26] and molecular studies [22].

A comparison of data sets A and B reveals that increasing the number of genes from 10 to 21 can improve bootstrap support values, a result consistent with other studies (e.g. [1-5]). In contrast, increasing the number of taxa (comparison of data set A and C) depressed branch support for higher groups (e.g. G.B.R.Y. clade). Therefore, if the goal is to achieve strong branch support for deep divergences, it may be favorable to focus on sequencing many genes for fewer taxa. However, sequencing many characters for few taxa can lead to problems such as long-branch attraction [76,77] and produce relationships that are misleading (e.g. [78-81]). Thus, if the researcher is faced with limited resources and seeks to improve support for deep divergences without introducing misleading artifacts, a solution may be to partially augment more characters for a selected but broad taxonomic sample. In our study, a deep divergence that was initially weak in support (the G.B.R.Y. clade) now has stronger branch support and the relationships are largely consistent with prior morphological hypotheses. While we have chosen a broad taxonomic sample to augment additional characters, future studies should examine how the choice of taxa for augmentation can influence the support for the correct tree.

### Synonymous changes and base compositional heterogeneity

Overall, two non-synonymous characters sets, degen1 and amino acids, provided the highest support for deep-level relationships in Gracillarioidea. These analyses resulted in a monophyletic G.B.R.Y. clade, for which support in some cases was very robust. In contrast, nt123, the codon model and nt123 partitioned analyses provided little or no support for deep relationships among gracillarioid families. When synonymous sites are added, only weak signal (≤ 62% BP) remains for the G.B.R.Y. clade. We speculate that analyses that include synonymous changes, even when that signal is down-weighted or modeled separately, do not effectively correct for the strong compositional heterogeneity found at nt3, leaving that non-phylogenetic signal to conflict with and obscure the true signal from non-synonymous change.

Strong compositional heterogeneity can incorrectly group unrelated taxa together [56,57], or equivalently, widely separate a taxon with strongly divergent base composition from its true relatives. Under the three character treatments, nt123, codon model, and nt123 partitioned, *Bucculatrix *(Bucculatricidae) was placed between the Tineidae and the remaining taxa (Additional files 4, 5, 6), a position difficult to reconcile with morphology. Compositional heterogeneity may account for the strikingly different placement of Bucculatricidae. Because non-synonymous changes strongly support the monophyly of the G.B.R.Y. clade, synonymous changes, mostly at nt3, must account for the less decisive placement of Bucculatricidae in nearly all nt123 trees. These results support our previous findings (e.g. [21]) that filtering synonymous substitutions (and thereby compositional heterogeneity) can result in more robust phylogenetic inference at deep levels.

A comparison of the ML topology with the neighbor-joining GTR ML distance and with Euclidean compositional distance trees for nt123 and nt3 suggests that the uncertain placement of Bucculatricidae in the nt123 data set is largely due to nt3 (Additional file 3). In the compositional distance trees, six taxa (*Bucculatrix sp., Atteva punctella*, *Eumetriochroa hederae*, *Hemerophila felis*, *Phyllocnistis citrella*, and *P. magnoliella*) fall between the Tineidae and the remaining taxa in a long internal branch. In the nt123 ML tree, in contrast, all taxa but *Bucculatrix *move to parts of the tree that are generally well supported and expected based on morphology and existing classifications (e.g. [11,35,82], *Eumetriochroa *with *Phyllocnistis*, and *Atteva *with *Eucalantica*).

Results of the ML nt3 analysis are very different, providing further evidence that compositional heterogeneity can affect trees based on nt3 alone. Despite providing about 90% of the total character change, the nt3 character set alone yields > 50% BP for only 7 nodes as compared to the full data set (nt123; 15 supported nodes), fewer even than the degen1 character set (13 supported nodes). Some unexpected relationships are found, such as *Bucculatrix *+ *Eumetriochroa*, which break up well-supported groups, in this case the monophyletic Gracillariidae (Additional file 3, F).

### Phylogenetic relationships of Gracillariidae and Gracillarioidea

Our results provide some of the first molecular evidence on the composition of and relationships within Gracillariidae and Gracillarioidea *sensu *Davis and Robinson [11]. Some previous hypotheses about those relationships were confirmed, and several novel ones proposed. Because rate variation between synonymous and non-synonymous sites was dramatic in the present study (see Table 3), we focus our discussion on the degen1 analyses unless otherwise noted. The discussion focuses on the degen1 tree from data set D (Figure 2) because it includes the most number of taxa and characters.

Gracillarioidea *sensu *Davis and Robinson [11], i.e. including Douglasiidae, was polyphyletic in all analyses, and monophyly of the superfamily was rejected significantly in six of eight AU tests (Table 4). Recently, Mutanen et al. [22] came to a similar conclusion based on fewer gracillarioid taxa and genes. In their analyses, Gracillarioidea were never monophyletic, and Douglasiidae was consistently placed in Apoditrysia. Mutanen et al. [22] also had difficulty in placing Bucculatricidae, which was paraphyletic with respect to *Tritymba *(Plutellidae), and Bucculatricidae + *Tritymba *was sister to Gracillariidae with weak (< 50%) ML bootstrap support. Based on our study and Mutanen et al. [22], Gracillarioidea as currently defined, will need to be reevaluated. Future studies should also sequence the genes included in the present study for *Ogmograptis *(Bucculatricidae), a genus that could not be obtained.

To resolve a possible sister group of Gracillariidae remains a challenging problem. Comparing ML trees from all analyses, Gracillariidae was most often closely related to Roeslerstammiidae + Yponomeutidae (the latter relationship which is congruent with morphology [83,84]). The close relationship of Yponomeutidae to Gracillarioidea (excluding Douglasiidae) is also consistent with other molecular studies [7,21,22]. These reports suggest, at least tentatively, that the putative morphological apomorphies proposed for Gracillarioidea by Davis and Robinson [11] may be homoplasies. In order to restore monophyly of the superfamily, we would need to exclude Douglasiidae from Gracillarioidea or include Yponomeutidae. However, more convincing resolution of inter-family relationships is desirable before any formal taxonomic changes are made.

Monophyly of Gracillariidae was strongly supported in nearly all analyses, a relationship that is corroborated by at least two morphological synapomorphies [26]. The grouping of Oecophyllembiinae (*sensu *Kumata) + Phyllocnistinae, which share unique serpentine mine morphology [11,66] and a highly specialized spinning instar [17], was supported weakly or not at all in our analyses. However, this sister-group relationship could not be rejected by any AU test, and this grouping was well supported by *Period *(82% BP, Additional file 2). The sister-group relationship of Gracillariinae to Lithocolletinae proposed by Kuznetzov and Stekol'nikov [24] was rejected by four of eight AU tests (Table 4). Our results strongly support the inclusion of *Leucanthiza *in Lithocolletinae, suggesting that this genus should be transferred from Gracillariinae, a conclusion that is also supported by larval morphology (Wagner and Davis unpubl. data). Monophyly of Gracillariinae (both with and without *Leucanthiza*) was rejected by the AU test in more than half of the data sets, suggesting that this subfamily needs to be revised. However, we did identify four genus-level groups with strong support within Gracillariinae: *Acrocercops*, *Gracillaria*, *Parectopa *groups and *Dendrorycter + Marmara*, all which were previously postulated based on morphology and life-history comparisons [18,69-72].

## Conclusions

Several main conclusions can be drawn from this study. First, branch support was maximized when gene sampling was increased, especially when we analyzed only the non-synonymous changes. Second, augmenting a taxon-rich data set (data set C; 57 taxa × 10 genes) with additional sequence data for approximately half the taxa substantially increased deep node support in a resource-efficient manner, apparently without inducing phylogenetic artifacts due to large blocks of missing data. While these two conclusions were drawn specifically from the data sets in this study, they are congruent with the results of Cho et al. [7]. Third, Gracillariidae were monophyletic in all our analyses, and nearly all species can be placed into one of six strongly supported clades, though relationships among them remain largely unclear. Fourth, Gracillarioidea, as defined by Davis and Robinson [11], clearly do not include Douglasiidae, and changes to the classification will be required. Fifth, the difficulty in placing Bucculatricidae is probably attributable to base compositional heterogeneity at nt3. From our tests for compositional heterogeneity and strong bootstrap values obtained when synonymous changes are excluded, we tentatively conclude that Bucculatricidae is closely related to Gracillariidae + Roeslerstammiidae + Yponomeutidae. Finally, the different levels of branch support seen under our different character treatments reinforce the importance of assessing confidence in groups under multiple phylogenetic approaches. Factors such as compositional heterogeneity, which can influence phylogenetic accuracy, are most easily assessed when data are partitioned into largely non-synonymous and mostly synonymous character sets. Branch support overall is strongest when all changes are included, but for several deep divergences, strong support is obtained only when synonymous changes were excluded. Because branch support for many deep splits was weak, we are exploring whether greater branch support for gracillariids and relatives can be achieved by means of genomic (next-generation) sequencing -- the focus of a future project.

## Authors' contributions

AYK carried out the RT-PCR experiments, sequence alignment, phylogenetic analyses, and drafted the manuscript. IO and AK conducted PCR work on two genes, H3 and EF-1α. JCR and CM provided funds for conducting the molecular work and helped design the study. MPC provided phylogenetic programs and hardware through the Lattice Project. AK, DRD, DLW, IO, JDP, and CLV collected valuable specimens necessary for the project. All authors read and approved the final manuscript.

## Supplementary Material

Additional file 1 **Exemplar species included, their classification, and GenBank accession numbers**. For Gracillariidae the number of taxa in each subfamily and genus is listed in parentheses (number of taxa sampled/number of taxa known). "x" denotes a sequence that could not be amplified. Click here for file

Additional file 2 **Single gene bootstrap values for all nodes in the nt123 tree of data set B**. Shaded boxes are those with > 80% bootstrap support. "ALL" refers to dataset B (all genes included). See Additional file 1 for taxon code names. Click here for file

Additional file 3 **Comparison of Euclidean compositional distance (NJ), GTR ML distance (NJ), and ML trees for nt123 and nt3**. Arrows indicate a long internal branch in the Euclidean compositional distance trees. Click here for file

Additional file 4 **Maximum likelihood nt123 trees for data sets A through D**. Scale bar = 0.07 substitutions/site. Click here for file

Additional file 5 **Maximum likelihood trees based on a codon model**. Scale bar = 0.03 substitutions/site. Click here for file

Additional file 6 **Maximum likelihood trees based on a partitioned model**. Scale bar = 0.2 substitutions/site. Click here for file

Additional file 7 **Maximum likelihood trees based on inferred amino acids**. Scale bar = 0.03 substitutions/site. Click here for file
